# Factors Associated With Delayed Presentation for Myocardial Infarction Among Patients in Iraq

**DOI:** 10.7759/cureus.104684

**Published:** 2026-03-04

**Authors:** Mustafa Al Jaafar, Muataz M Naeem, Noor S Mandalawi

**Affiliations:** 1 Internal Medicine, Ibn Zuhur Hospital, Baghdad, IRQ; 2 General Surgery, Baghdad Teaching Hospital, Baghdad, IRQ; 3 General Surgery, Martyr Sadr Hospital, Baghdad, IRQ

**Keywords:** acute coronary syndrome, delayed presentation, dyslipidemia, myocardial infarction, risk factors

## Abstract

Background

Myocardial infarction (MI) is a leading cause of morbidity and mortality worldwide, characterized by myocardial necrosis due to acute coronary artery obstruction. Early hospital presentation optimizes reperfusion therapy and reduces complications, yet delays persist, especially in low- and middle-income countries like Iraq.​

Patients and methods

This cross-sectional retrospective study was conducted in Baghdad, Iraq, from April to October 2025 and included 150 adult patients with acute MI presenting to the emergency department. Data from medical records assessed demographics, clinical factors, and presentation timing (delayed if >12 hours’ post-symptom onset). Fisher's exact test identified associations (p<0.05 significant).​

Results

Of 150 patients, 59 (39.3%) presented early (≤12 hours) and 91 (60.7%) presented late (>12 hours). Delayed presentation was significantly associated with older age (≥55 years; p=0.014, OR=2.59, 95% CI=1.24-5.40), diabetes mellitus (p=0.025, OR=2.42, 95% CI=1.17-5.01), dyslipidemia (p=0.013, OR=2.49, 95% CI=1.24-5.01), and nighttime symptom onset (12 AM-8 AM; p=0.008, OR=2.50, 95% CI=1.28-4.91). No significant associations emerged for gender (p=0.735), BMI (p=0.464), education (p=0.664), residency (p=0.729), hypertension (p=0.467), family history (p=0.650), or prior percutaneous coronary intervention (p=0.721).​

Conclusion

Over 60% of Iraqi MI patients presented later than 12 hours after symptom onset, with delayed presentation linked to older age, diabetes, dyslipidemia, and nighttime onset. Addressing these via targeted education could enhance outcomes.

## Introduction

Myocardial infarction (MI), or heart attack, is defined as myocardial necrosis resulting from an acute obstruction of blood flow in a coronary artery, causing ischemia and death of heart muscle tissue. MI is a type of acute coronary syndrome typically presenting with chest discomfort, but symptoms can vary widely. MI is myocardial cell death due to prolonged ischemia, confirmed by a rise and/or fall of cardiac biomarkers (preferably troponin) above the 99th percentile of the upper reference limit.

Along with biomarker evidence, at least one of the following must be present for diagnosis: symptoms of ischemia (e.g., chest pain, shortness of breath), new ECG changes indicative of ischemia (ST segment elevation or depression, new left bundle branch block), development of pathological Q waves on ECG, imaging evidence of new loss of viable myocardium or new regional wall motion abnormality, and identification of intracoronary thrombus by angiography or autopsy [1,2].

Cardiovascular disease is a prominent source of morbidity and mortality worldwide, representing around 31% of all fatalities [3]. Ischemic heart disease, including acute MI, is the principal cause of these deaths, with its frequency on the increase [4]. On a worldwide scale, acute MI impacts around 15.9 million persons annually [5], with over three million categorized as ST-segment elevation MI [6].

MI prevalence varies worldwide but is estimated at about 3% in adults aged 20 and over in the United States, with millions affected annually [6]. In the United States, about 16.5 million adults live with coronary artery disease, which predisposes to MI, with approximately 605,000 new MI cases and 200,000 recurrent cases annually [7]. MI incidence increases with age and is generally higher in men than women, but mortality affects both sexes significantly [8].

Globally, delayed MI presentation (>12 hours) heightens complications such as left ventricular thrombus (5-15% incidence in late STEMI, exacerbated by stagnation and injury) [9], cardiogenic shock with 77.3% in-hospital mortality, 81.8% major adverse events, and elevated acute kidney injury (72.7%) in late versus early presenters [10]. De Luca et al. in a large primary angioplasty cohort showed that each 30-minute delay raised one-year mortality by 7.5% [11]. A registry of 348 acute coronary syndrome patients in Baghdad's Medical City Complex found 65% of STEMI cases presented ≥12 hours after symptom onset, with a median time of 11.5 hours. Only 15% arrived <6 hours, linked to poor symptom recognition, and in-hospital mortality reached 7.7%, higher than many global registries due to delays and limited percutaneous coronary intervention (PCI) [12]. A 2021 systematic review by Beza et al. across low- and middle-income countries reported mean symptom-to-treatment delays far beyond optimal windows, with 66.1% presenting >6 hours in one cohort, leading to heart failure as the top complication, low reperfusion rates (e.g., 14.9% STEMI mortality), and poorer outcomes compared to high-income settings [13].

Early presentation in MI cases is critically important as it allows timely reperfusion therapy, which significantly reduces myocardial damage and improves patient outcomes. Research shows that the shorter the ischemic time - from symptom onset to treatment - the lower the risk of complications such as heart failure, arrhythmias, and death. A study analyzing ST elevation MI patients found that early presenters had fewer complications and better survival rates, with health awareness and education strongly linked to earlier emergency room arrival. Delays beyond 4-6 hours increase the risk of no-reflow phenomenon after angioplasty and higher mortality. Thus, early recognition of symptoms and prompt medical intervention is essential to maximize recovery and minimize the burden of MI complications [10,14,15].

Patient awareness is a key factor in improving delay. Public awareness campaigns have demonstrated effectiveness in reducing prehospital delays for MI patients by enhancing symptom recognition and prompting faster medical seeking. An Australian study found that awareness of the Heart Foundation's warning signs campaign was linked to shorter decision times and prehospital delays [16]. An Iranian field trial with 131 MI patients showed SMS education on symptoms and urgency reduced mean onset-to-door time from 291.7 to 240.5 minutes, with odds of arriving within 120 minutes rising 5.8-fold due to quicker calls for help [17].

Therefore, understanding and addressing the risk factors of delayed MI through specific public health interventions is key to minimizing delays in MI care and reducing the risk of complications and mortality [18].

## Materials and methods

This cross-sectional retrospective study was conducted in Baghdad, Iraq, during the period of April 2025 to October 2025. The study population comprised patients who presented to the emergency department and were subsequently admitted with a primary diagnosis of acute MI.

Inclusion criteria

The inclusion criteria were adult participants aged 18 years or older at the time of admission with confirmed symptomatic acute MI based on established clinical and laboratory criteria.

Exclusion criteria

The study excluded patients with incomplete, illegible, or missing information critical to the study variables (e.g., time of symptom onset, key co-morbidities); patients with severe communicative issues (such as profound hearing impairment or documented severe cognitive disorders) that prevented accurate history collection by the attending medical staff at the time of presentation (as noted in the initial medical record); patients with severe, confounding systemic diseases, including New York Heart Association (NYHA) class IV heart failure, end-stage renal failure requiring dialysis, or active malignancy. They were excluded to isolate MI-specific delays.

Ethical consideration

Written informed consent was obtained from patients before collecting the data. An official letter of approval was obtained from an Institutional Review Board prior to data collection.

Sample and data collection

A total of 150 patients diagnosed with MI were included in the studied sample. Data collection for this research was conducted through a retrospective review of patient history records documented at the hospital. Convenient sampling was used. Data extraction used a standardized form by one trained reviewer, with 10% of records validated by a second reviewer (98% agreement). Adult patients diagnosed with acute MI were identified from hospital admission logs over the study period.

MI diagnosis was based on the American College of Cardiology/American Heart Association guidelines, which are freely available clinical guidelines [19]. This required evidence of myocardial injury (rise and/or fall of cardiac troponin values) with clinical evidence of acute myocardial ischemia (e.g., ischemic symptoms, new ECG changes). Patient demographic data (age, sex, BMI, educational level, residency) and clinical variables (presence of diabetes mellitus, hypertension, dyslipidemia, family history of cardiovascular disease, previous PCI) were extracted retrospectively from hospital medical records. Symptom-onset time was reported by patients in initial emergency department interviews.

Definition of presentation delay

The critical variable of time to presentation was calculated as the interval between the documented time of symptom onset (as recorded during the initial patient interview in the emergency department) and the exact time of arrival at the hospital. For the purpose of this study, delayed MI presentation was defined as a patient’s arrival at the emergency department >12 hours from the onset of symptoms. Patients arriving at ≤12 hours were classified as the early presentation group.

Data entry and analysis

Data entry was performed using Microsoft Excel 2019 (Microsoft Corp., Redmond, WA), and statistical analyses were conducted with SPSS Version 26 (IBM Corp., Armonk, NY). Categorical variables were presented as frequencies and percentages. Fisher’s exact test was used to analyze associations between clinical and demographic factors and early versus late presentation. A p-value of ≤0.05 was considered statistically significant.

## Results

**Figure 1:**
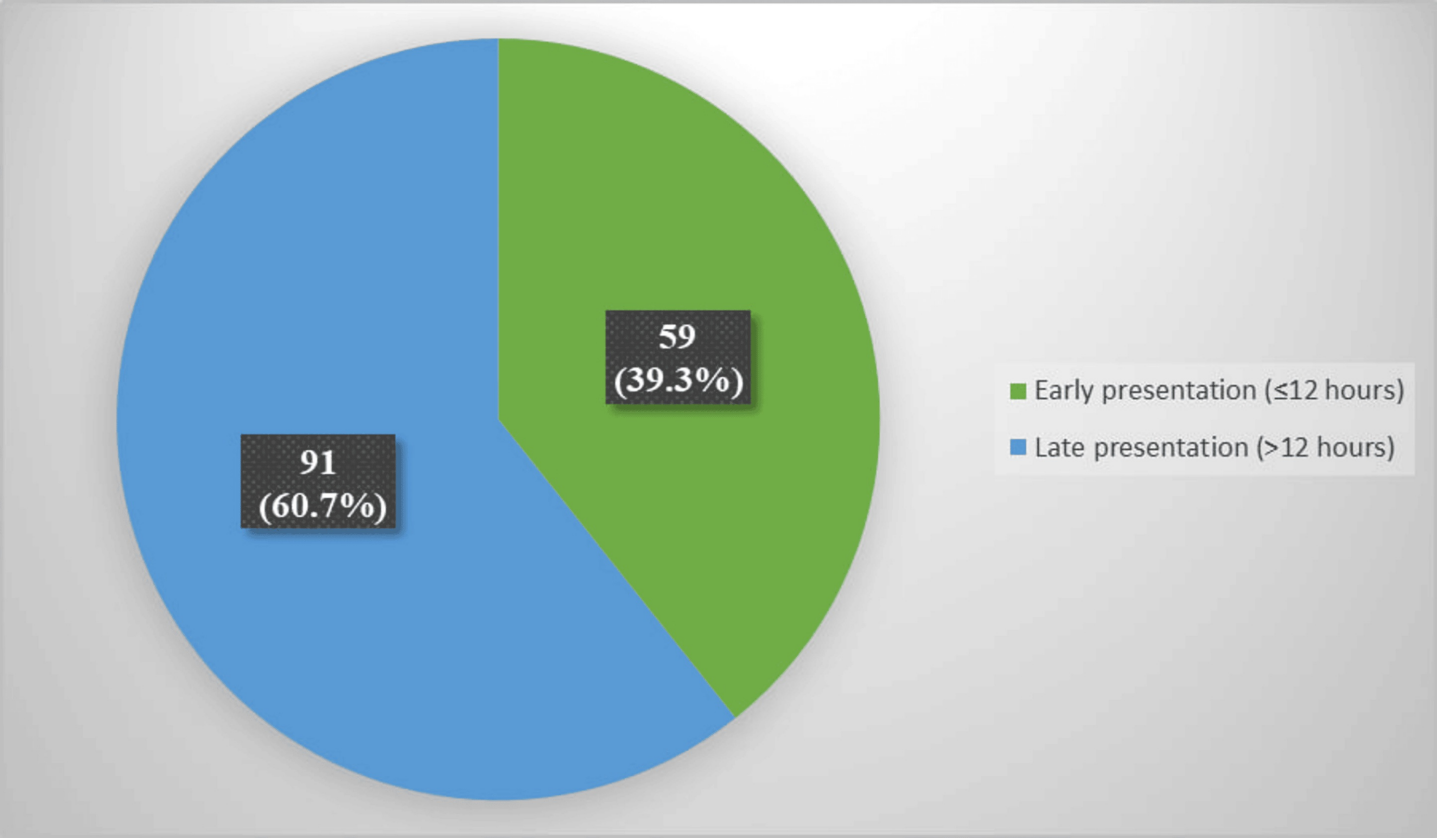
Distribution of the studied sample according to the time of presentation. Data are presented as frequencies and corresponding percentages (%).

A total number of 150 patients with MI were included in the study sample. Among 150 patients, 59 (39.3%) presented early, whereas 91 (60.7%) presented late (Figure 1).

Of the 150 patients studied, 59 (39.3%) presented early (≤12 hours) and 91 (60.7%) presented late (>12 hours) (Table 1). Age was significantly associated with delay; patients aged ≥55 years were more likely to present late (73/109, 67.0%) compared to those <55 years (36/109, 33.0%; p=0.014, OR 2.59, 95% CI 1.24-5.40). No significant differences were observed for gender (p=0.735), BMI categories (p=0.464), educational levels (p=0.664), or residency (p=0.729). Specifically, 59.1% of males and 62.9% of females presented late.

**Table 1:** Association between basic characteristics and time to emergency presentation. Odds ratios and 95% confidence intervals are calculated with the respective reference groups as indicated. An OR of >1 indicates increased odds of late presentation, while an OR of <1 indicates decreased odds of late presentation, relative to the reference group.

Basic characteristics	Early presentation (≤12 hours), N = 59	Late presentation (>12 hours), N = 91	P-value	Odds ratio (95% CI)
Age (years)				
<55	23 (56.1%)	18 (43.9%)	0.014	Reference
≥55	36 (33.0%)	73 (67.0%)	-	2.59 (1.24-5.40)
Gender				
Male	36 (40.9%)	52 (59.1%)	0.735	Reference
Female	23 (37.1%)	39 (62.9%)	-	1.17 (0.60–2.29)
BMI (kg/m^2^)				
Underweight (<18.5)	1 (50.0%)	1 (50.0%)	0.464	0.47 (0.03–8.01)
Normal weight (18.5–24.9)	17 (32.1%)	36 (67.9%)	-	Reference
Overweight (25.0–29.9)	39 (42.4%)	53 (57.6%)	-	0.64 (0.32–1.30)
Obese (≥30.0)	2 (66.7%)	1 (33.3%)	-	0.24 (0.02–2.79)
Educational level				
Primary school	19 (41.3%)	27 (58.7%)	0.664	Reference
High school	21 (35.0%)	39 (65.0%)	-	1.31 (0.59–2.88)
College	19 (43.2%)	25 (56.8%)	-	0.93 (0.40–2.14)
Residency				
Urban	36 (37.9%)	59 (62.1%)	0.729	Reference
Rural	23 (41.8%)	32 (58.2%)	-	0.85 (0.43–1.67)

Significant associations with delayed presentation were found for diabetes mellitus (72/108 late, 66.7%; p=0.025, OR 2.42, 95% CI 1.17-5.01), dyslipidemia (68/100 late, 68.0%; p=0.013, OR 2.49, 95% CI 1.24-5.01), and nighttime symptom onset between 12 AM and 8 AM (56/79 late, 70.9%; p=0.008, OR 2.50, 95% CI 1.28-4.91) (Table 2). Conversely, there was no significant association with hypertension (p=0.467), family history of ischemic heart disease (p=0.650), or previous PCI (p=0.721).

**Table 2:** Association between clinical characteristics and time to emergency presentation. Odds ratios and 95% confidence intervals are calculated with the respective reference groups as indicated. An OR of >1 indicates increased odds of late presentation, while an OR of <1 indicates decreased odds of late presentation, relative to the reference group.

Clinical characteristics	Early presentation (≤12 hours)	Late presentation (>12 hours)	P-value	Odds ratio (95% CI)
Diabetes mellitus				
Yes	36 (33.3%)	72 (66.7%)	0.025	2.42 (1.17–5.01)
No	23 (54.8%)	19 (45.2%)	-	Reference
Hypertension				
Yes	39 (37.1%)	66 (62.9%)	0.467	1.35 (0.67–2.75)
No	20 (44.4%)	25 (55.6%)	-	Reference
Dyslipidemia				
Yes	32 (32.0%)	68 (68.0%)	0.013	2.49 (1.24–5.01)
No	27 (54.0%)	23 (46.0%)	-	Reference
Family history of ischemic heart disease				
Yes	8 (33.3%)	16 (66.7%)	0.65	1.36 (0.54–3.41)
No	51 (40.5%)	75 (59.5%)	-	Reference
Previous history of PCI				
Yes	17 (37.0%)	29 (63.0%)	0.721	1.16 (0.57–2.36)
No	42 (40.4%)	62 (59.6%)	-	Reference
Symptoms onset at night (12-8 AM)				
Yes	23 (29.1%)	56 (70.9%)	0.008	2.50 (1.28-4.91)
No	36 (50.7%)	35 (49.3%)	-	Reference

## Discussion

The American Heart Association defines delay in MI presentation primarily in terms of the time interval from symptom onset to first medical contact. Patient delay refers to the duration between the onset of MI symptoms and the decision to seek medical care, which constitutes the largest portion of prehospital delay. Official guidelines emphasize that the total ischemic time should be minimized, ideally within 12 hours, to maximize the benefit of reperfusion therapies. Prolonged patient delay is known to increase morbidity and mortality due to missed opportunities for early intervention [19].

An unfortunate finding in our study is that only 39.3% of patients presented early to the emergency department. This is in concordance with a study in Baghdad, which reported that among 160 patients, 70 (43.8%) had delayed presentation (>12 hours) [20]. A systematic review in low- to middle-income countries found that the mean time from symptom onset to first medical contact was around 12.7 hours, ranging widely from 10 minutes to 96 hours [13]. The shortfalls in timely presentation of MI patients to the emergency department could be attributed to an underdeveloped emergency transport infrastructure and inadequate communication and organization between community and interventional centers, leading to delays in acute coronary syndrome treatment.

The present study found that older age (≥55) was a significant risk factor for delay MI presentation (OR: 2.59). This is similar to the study by Taghaddosi et al., who found that old age was significantly associated with delay MI presentation [21]. It is suggested that older adults have an increased risk of atypical symptoms, comorbidities, and social factors such as living alone and reduced mobility, leading to poorer symptom recognition and slower care-seeking [22].

The current study found that diabetes mellitus was significantly associated with delay MI presentation (OR: 2.42). This is in concordance with the study by Alidoosti, who found that diabetes was significantly associated with increased risk of delay MI presentation (OR: 1.89) [23]. Diabetes may contribute to atypical MI symptoms due to diabetic neuropathy, which impairs sensory nerve function, resulting in reduced chest pain and altered symptom perception. This neuropathy, combined with complex coronary disease and comorbidities, leads to less typical symptoms such as shortness of breath or fatigue, causing delays in recognizing and seeking care for MI [24].

The present study found that dyslipidemia was significantly associated with delay MI presentation (OR: 2.49). A multivariant study by Albrahim et al. found that dyslipidemia patients had a four times greater risk of late presentation, with prevalence of dyslipidemia of 54.2% among MI patients who presented late in the studied sample [25]. Dyslipidemia is associated with atherosclerosis, which gradually narrows the coronary arteries and leads to chronic ischemia before an acute event occurs. This slow progression can mask symptoms, causing patients to present later after symptom onset [26].

The current study found that symptoms onset at night (12-8 Am) was significantly associated with delay MI presentation (OR: 2.5). A study by Alidoosti found that symptoms onset at night was significantly associated with a two-fold increased risk of delayed MI presentation [23]. Patients are less likely to recognize or respond to symptoms at night, and there is often reduced access to immediate medical care or slower emergency response during night hours. Additionally, nighttime symptoms might cause hesitation or confusion about severity, further prolonging time to hospital presentation [27].

Study limitations

While guidelines emphasize minimizing total ischemic time, our cross-sectional data identify associations, not causation. Multivariate analysis was not conducted, precluding adjustment for potential confounders and strengthening of these associations. Documentation bias likely affected symptom onset times, especially in older adults (atypical symptoms) and diabetics (neuropathy masking pain), inflating perceived delays. Exclusion of severe comorbidities (e.g., NYHA class IV heart failure, dialysis) may enhance internal validity but reduces generalizability to broader Iraqi MI populations. Moreover, associations such as older age, diabetes, dyslipidemia, and nighttime onset with delays may be influenced by unmeasured factors such as limited transport availability, low socioeconomic status, and poor health literacy - common in Iraqi and LMIC contexts. These could prolong decision-to-seek-care times, independent of measured demographics.

## Conclusions

In this cross-sectional retrospective study of 150 Iraqi MI patients, unadjusted analyses identified significant associations between delayed presentation (>12 hours), and older age (≥55 years), diabetes mellitus, dyslipidemia, and nighttime symptom onset (all p<0.05). Overall, 60.7% of patients presented late. Public awareness campaigns emphasizing atypical symptoms in diabetes/older age, SMS-based education, and enhanced nighttime ambulance access could address identified associations. Prospective, multivariable studies are needed to validate these strategies and confirm their impact on outcomes in Iraq's context.
